# Association Between Type 2 Diabetes and Viruses with Oncomodulatory Activity in Patients with Squamous Cell Carcinoma

**DOI:** 10.3390/cimb48060560

**Published:** 2026-05-27

**Authors:** Ivo Nikolaev Sirakov, Kalina Shishkova, Raina Gergova, Stefan Dimitrov Gergov, Elena Tasheva-Terzieva

**Affiliations:** 1 Department of Medical Microbiology “Corr. Mem. Prof. Ivan Mitov, MD, DMSc”, Faculty of Medicine, Medical University of Sofia, 2 “Zdrave” Str., 1431 Sofia, Bulgaria; rtgergova@gmail.com; 2 Laboratory of Virology, Faculty of Biology, University of Sofia “St. Kl. Ohridski”, 1164 Sofia, Bulgaria; k_shishkova@biofac.uni-sofia.bg; 3 Department Head and Neck Surgery, National Oncological Hospital “Ivan Chernozemski”, 1504 Sofia, Bulgaria; s_gergov@mail.bg; 4 Department of Zoology and Anthropology, Faculty of Biology, University of Sofia “St. Kl. Ohridski”, 8 Dragan Tzankov Blvd., 1164 Sofia, Bulgaria; elena.tasheva@gmail.com

**Keywords:** external auricle, head and neck cancer, oncogenic viruses, Epstein–Barr virus, Human papillomavirus

## Abstract

Type 2 diabetes is a multifactorial metabolic disease characterized by chronic hyperglycemia, insulin resistance, and persistent low-grade inflammation. All of these factors lead to dysregulation of the immune system. Of particular interest is the interaction between immune dysregulation in type 2 diabetes and oncogenic viruses such as Human papillomavirus (HPV) and Epstein–Barr virus (EBV), which play an essential role in the etiology of Head and neck cancer on the one hand and have mechanisms for escaping the immune response on the other. The aim of the present study is to perform an analysis of patients with head and neck cancer divided into two groups, with and without diabetes, aimed at studying the relationship between type 2 diabetes and the established viral status. It was found that for all viruses proven by us, the frequency of positive tests for them was higher in the group with type 2 diabetes compared to the group of patients without diabetes. The study provides new insights and suggestions for a significant association between type 2 diabetes mellitus, increased prevalence of EBV, and some low-risk HPV genotypes in patients with head and neck tumors. Continuing from our previous study, the association between EBV and HPV44, after strict statistical adjustment, highlights their potential biological and clinical significance within the oncogenic environment in the presence of type 2 diabetes.

## 1. Introduction

Type 2 diabetes is a chronic metabolic disease characterized by persistent hyperglycemia, insulin resistance, and systemic metabolic dysregulation [1,2]. In addition to established cardiovascular and microvascular complications, this type of disease is associated with immune system dysregulation, chronic inflammation, and increased susceptibility to infectious and neoplastic diseases. Epidemiological studies have shown that people with type 2 diabetes exhibit higher susceptibility to various malignancies associated with worse prognoses compared to non-diabetic patients. These data emphasize the role of diabetes as a factor influencing the development and progression of cancer [3,4].

Oncogenic viral infections are key factors in carcinogenesis in some types and forms of cancer, including those localized in the head and neck region [5,6]. Some viruses can facilitate malignant transformation through direct genetic alterations, chronic inflammation, or persistent immune activation. Epstein–Barr virus (EBV) and human papillomavirus (HPV) are particularly important in this context. EBV is etiologically associated with nasopharyngeal carcinoma (NPC) and has been identified in subsets of other head and neck malignancies. Other members of the herpesvirus family, such as cytomegalovirus (CMV) and herpes simplex virus type 1 (HSV-1), are found in clinical specimens in various neoplastic settings, but their presence is associated with the underlying immune status of the organism rather than with a direct oncogenic effect [7]. High-risk HPV genotypes are well-established etiological causes of oropharyngeal squamous cell carcinoma, cervical cancer, etc. (OPSCC) [5,8,9,10,11].

Head and neck cancer is a heterogeneous group of malignancies associated with different anatomical subregions, each of which exhibits distinct epidemiological and virological characteristics [5,11]. The majority of the literature on viral involvement in head and neck cancer has focused on nasopharyngeal and oropharyngeal carcinomas, where EBV and HPV predominate [8,12,13,14,15,16,17]. However, the prevalence of viruses and the patterns of infection can vary significantly in different regions of the head and neck [5,11,18]. Also, the interactions between viruses, coinfections, and host factors, such as metabolic diseases, remain incompletely understood across the broader spectrum of head and neck malignancies.

Type 2 diabetes (DM2) may modulate the development and progression of some viral infections, as well as their persistence, through multiple mechanisms, including impaired innate and adaptive immunity, chronic inflammation, oxidative stress, and altered cytokine milieu [12,19,20,21]. Several authors have reported an increased incidence of infections and reactivation of latent viruses in people with diabetes, suggesting that metabolic dysregulation provides a permissive environment for viral persistence. In the field of oncology, DM2 is associated not only with the observed increased incidence of cancer, but also with more aggressive disease phenotypes and worse prognosis, for example, in head and neck cancers [3,4,22,23,24]. Despite these associations, most studies have focused on the risk of developing cancer, response to treatment, or patient survival [24,25,26,27], but the focus on patterns of viral infections within people with cancer and in the presence of a disease such as type 2 diabetes has been somewhat neglected.

Relatively few studies have attempted to establish a direct or indirect correlation of DM with the dynamics of the spread of oncogenic and/or oncomodulatory viruses among patients with already diagnosed head and neck cancer [20,28]. Even more limited are the data for studying and assessing the simultaneous presence of multiple viral agents in coinfection and their possible association with DM2 [5,8,15]. This gap is particularly evident with regard to low-risk HPV genotypes, which are less frequently studied compared to high-risk ones, but may contribute to the overall picture in patients affected by type 2 diabetes. Furthermore, the potential interaction between type 2 diabetes and patterns of viral coinfection remains largely unexplored.

Available data suggest possible links between DM2, viral infection, and carcinogenesis, involving impaired immune surveillance, mitochondrial dysfunction, enhanced viral persistence, and chronic inflammatory signaling [19,20,27,28,29]. However, these frameworks are based on data obtained primarily at the population level or from experimental models and have not been systematically evaluated in small, well-characterized clinical groups of people with relatively detailed viral profiling. On the other hand, other authors have not found such a link or consider it to be insignificant [30,31,32]. Some authors have found an inverse relationship between diabetes and the development of head and neck cancer, most likely due to the use of metformin [32] and thiazolidinediones [33].

For all of the above, exploratory analyses integrating metabolic status with virological data in patients with head and neck cancer are warranted, using rigorous statistical approaches to mitigate overinterpretation. At this stage, there are no definitive data available demonstrating associations between type 2 diabetes and viral infections in patients with head and neck malignancies originating from anatomical sites other than the nasopharyngeal and oropharyngeal regions.

In our previous study, these 41 biopsy specimens obtained from patients with squamous cell carcinoma of the external auricle were examined for the presence of viruses with oncogenic and/or oncomodulatory activity. The obtained results showed that no high-risk HPV genotype was detected in any of the specimens. The highest percentage of samples showed genotype 6/11, and the lowest number of samples showed low-risk genotype 44. Of all herpes viruses, EBV was detected in the largest proportion of samples, present as a co-infection with HPV, and always together with genotype 6/11. In the other herpes viruses, the presence of HSV-2 was not confirmed in any of the samples. HSV 1 was present in only three of the samples, as a co-infection with genotypes 6/11, 42, and 43. When examining the samples for the presence of HCMV, only one positive sample was found, with both HPV 6/11 and 42 also present. The presence of the mentioned viruses, as well as the non-random distribution of EBV + HPV 6/11 and EBV + HPV 44, proven by us, does not necessarily make them etiological agents, but they could, through different and known mechanisms, influence the initiation and/or modulation of carcinogenesis (quote from our article). After a thorough study of the health status of the patients, it turned out that a significant part of them had type 2 diabetes. Due to everything mentioned so far, the aim of the present study is to perform an analysis of patients with head and neck cancer, divided into two groups with and without diabetes, aimed at studying the relationship between type 2 diabetes and the established viral status.

## 2. Materials and Methods

### 2.1. Samples and Analyses

The study included 41 biopsy materials from the external auricle of patients with proven SCC obtained from the National Oncological Hospital “Ivan Chernozemski”. The patients were predominantly men (38) over the age of 60, and only three women. The samples were divided into two groups: patients with type 2 diabetes—DM2 (27 samples) and a group of patients without type 2 diabetes—nDM2 (14 samples). DNA was obtained from the biopsy materials, which were examined for HPV, EBV, CMV, BK Polyomavirus, and HSVs, as described in our previous study [5].

### 2.2. Statistical Analyses

The statistical analysis was aimed at assessing the relationship between the presence of type 2 diabetes (DM2) and viral infections.

For each of the two groups (patients with DM2 and without diabetes, nDM2), the frequency of patients with a positive test for the respective virus was calculated, and the results were presented as the number and percentage of patients.

For comparison between independent groups (DM2 and nDM2) regarding the frequency of viral infections, the χ^2^ test was used, and for expected frequencies below 5, Fisher’s exact test was applied.

For the analysis of the association between type 2 diabetes and viral status, HPV 6/11, 42, 43, 44, and EBV were used. The other two herpes viruses were detected in a small percentage of the samples and were not statistically significant for the present study.

For the assessment of the association between diabetes status and viral infection in the same patients, McNemar’s test was used. For each virus, patients were divided into four categories: DM2 and V—patients with diabetes, positive for the respective virus;

DM2 only—patients with diabetes, negative for the respective virus;

V only—patients without diabetes, positive for the respective virus;

Neither—patients without diabetes and negative for the respective virus.

The McNemar’s test compares the number of patients in the DM2 only and V only categories, allowing to assess whether there is a statistically significant association between type 2 diabetes and viral infection.

Due to the multiple comparisons performed in the McNemar analysis, *p*-values were corrected using the Holm–Bonferroni method.

Additionally, a descriptive analysis of the distribution of patients with and without diabetes among virus-positive and virus-negative cases was performed to visualize and aid in the interpretation of the results.

Results were considered statistically significant at *p* ≤ 0.05 after correction for multiple comparisons. A brief explanation of the application of the test is presented in Table 1.

McNemar’s test ignores the frequencies a and d—when there is both a virus and diabetes, and when there is no virus and no diabetes. This test only analyzes the frequencies of DM2+, virus−, and DM2− virus+. Cells b and c are called a “discordant pair”. The *p*-value can be used to analyze what the result is—*p* ≤ 0.05 means that there is a statistically significant asymmetry between the frequencies b and c (i.e., they differ). In this case, it is interpreted that there is an association between the viral infection and diabetes, i.e., the virus is more common in the diabetic group or the virus is more common in the group without diabetes. With such a result, possible explanations for this association should be given, considering that statistics do not show a causal relationship.

*p* > 0.05 means that the frequencies b and c are symmetrical/equal, i.e., there is no evidence of asymmetry and the frequencies are “balanced”. The interpretation of such a result includes the fact that no association has been proven between diabetes status and the presence of a viral infection. It is important to note that this does not mean that there is no such association, only that it has not been proven. It is possible that there really is none or that it cannot be proven with these data—usually the possibility of interpretation in this case is related to the sample size, and in our case, it is not large.

## 3. Results

### 3.1. Distribution of Proven Viral Infections in Patients with Type 2 Diabetes and Patients Without Type 2 Diabetes

After testing 41 biopsy specimens for HPV, EBV, CMV, and HSVs in a previous study, it was found that 36 samples (87.8%) were positive for low-risk HPV, 17 samples (41.5%) were positive for EBV, 3 samples (7.3%) were positive for HSV1, and one sample (2.4%) was positive for HCMV. The samples were negative for high-risk HPV genotypes, HSV2, and BK polyomavirus (quote from our article).

**Figure 1:**
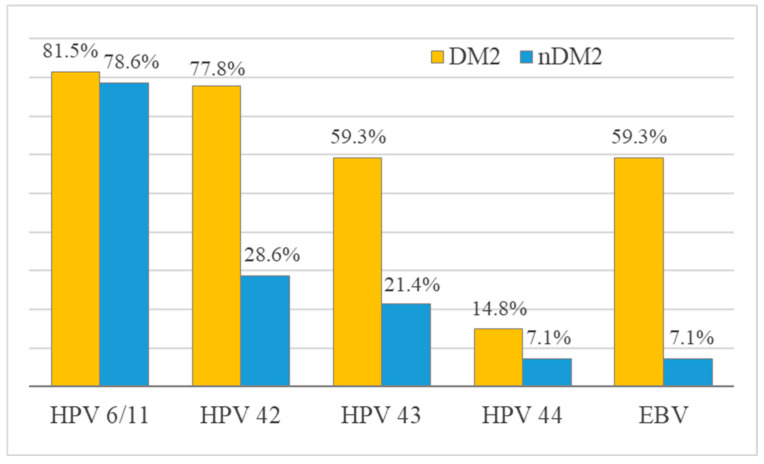
Comparison of viral prevalence between patients with and without type 2 diabetes mellitus (DM2).

When examining the prevalence of viral infections in both groups of patients, we found that for all viruses analyzed, the frequency of positive tests was higher in the group with type 2 diabetes compared to the group without diabetes. The largest differences between the two groups were observed for EBV (52.1 percentage points), HPV 42 (49.2 percentage points), and HPV 43 (37.9 percentage points). Statistically significant differences between the two groups were found for EBV (*p* = 0.004), HPV 42 (*p* = 0.006) and HPV 43 (*p* = 0.048), while no statistically significant differences were found for HPV 6/11, HPV 44, HSV-1 and CMV. The distribution of viral infections in the groups with and without type 2 diabetes is presented in Table 2 and Figure 1.

### 3.2. Association Between Type 2 Diabetes and Viral Status

McNemar’s test was used to assess the association between type 2 diabetes and established viral status in the same patients.

**Figure 2:**
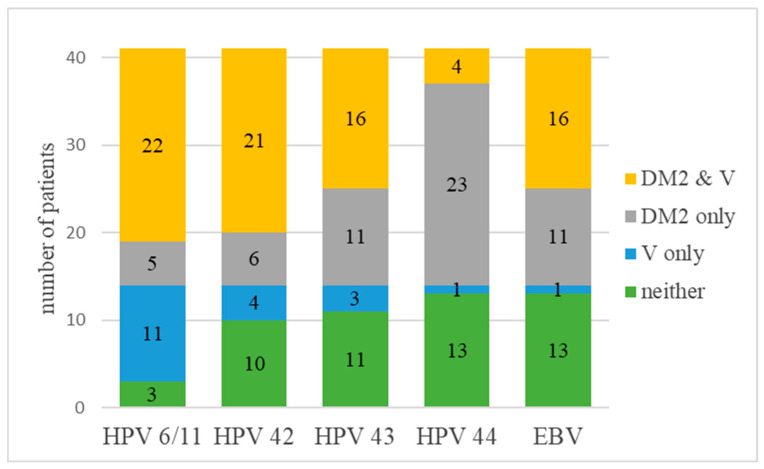
Distribution of co-infection patterns between type 2 diabetes mellitus (DM2) and viral positivity for HPV types and EBV.

The results of the analysis are presented in Table 3 and Figure 2.

After correction of *p*-values using the Holm–Bonferroni method, statistically significant associations were found for HPV 44 (adjusted *p* < 0.001) and EBV (adjusted *p* = 0.037), while no statistically significant associations were found for HPV 6/11, HPV 42, and HPV 43. For HPV 44 and EBV, there was a pronounced asymmetry between the discordant categories (DM2+, virus−) and (DM2−, virus+), indicating a non-random distribution. The result for EBV shows that the virus occurs at a significantly higher proportion in patients with diabetes compared to patients without diabetes, with the imbalance in the discordant pairs of cells supporting the presence of a statistically significant association.

### 3.3. Type 2 Diabetes Cases, in Percentage, Among Patients Positive and Negative for a Given Virus

**Figure 3:**
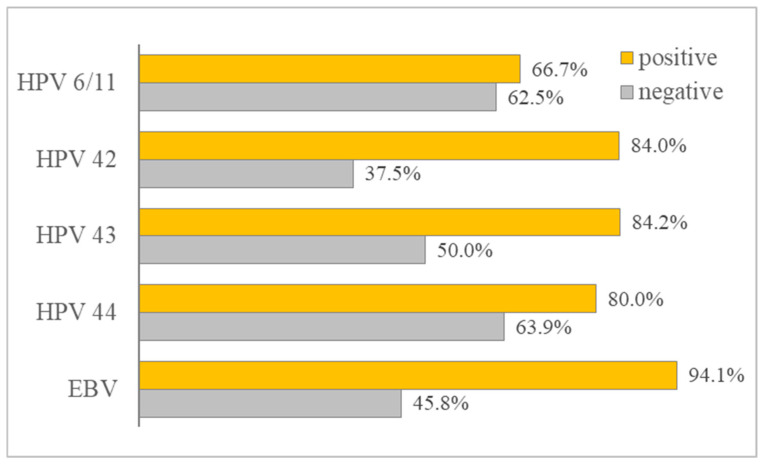
Percentage of patients with type 2 diabetes mellitus (DM2) among virus-positive and virus-negative groups for each virus.

**Figure 4:**
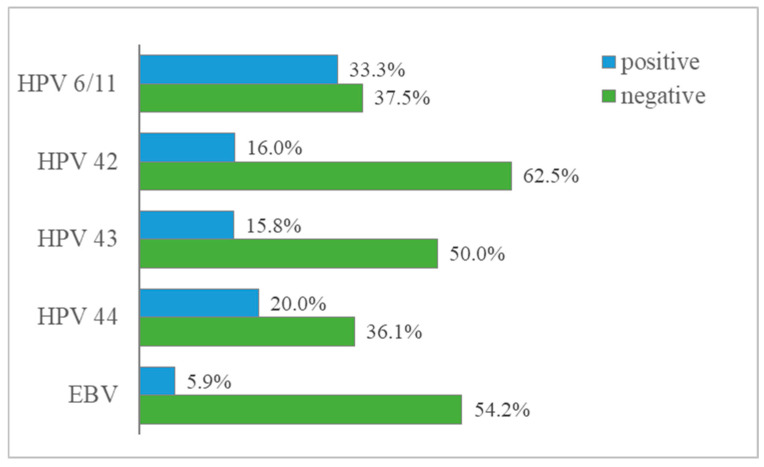
Percentage of non-diabetic patients (nDM2) among virus-positive and virus-negative groups for each virus.

Additional descriptive analyses of the distribution of diabetes status among virus-positive and virus-negative patients are presented in Figure 3 and Figure 4.

For each of the proven viruses, patients were divided into two groups: those with a positive and those with a negative test for the virus. The frequencies of diabetic patients in the two groups were calculated (Figure 3), as well as the frequency of patients without diabetes in the two groups, with a positive and a negative test for the respective viruses (Figure 4). The figures are only for the five viruses with a higher number of cases.

For all viruses analyzed, the proportion of patients with diabetes was higher among virus-positive than virus-negative patients, while the proportion of patients without diabetes was higher among virus-negative patients.

For EBV, among virus-positive patients, 94.1% (16/17) were diabetic and 5.9% (1/17) were non-diabetic. Among EBV-negative patients, 45.8% (11/24) were diabetic and 54.2% (13/24) were non-diabetic.

For HPV 44, among virus-positive patients, 80.0% (4/5) were diabetic and 20.0% (1/5) were non-diabetic. Among HPV 44-negative patients, 63.9% (23/36) were diabetic and 36.1% (13/36) were non-diabetic.

The observation of a higher percentage of viral infections in the diabetic group alone is insufficient, but a clear asymmetry between the discordant categories (DM2+/virus− versus DM2−/virus+) is necessary. Such asymmetry is clear for EBV and HPV 44, but is absent for the other viruses, which explains the difference in statistical significance.

When comparing EBV and HPV 44, the difference in EBV between % with diabetes in V+ and % with diabetes in V− is large: 94.1% and 45.8%, while this difference in HPV 44 is smaller: 80% and 63.9%, i.e., the effect is not as large.

McNemar’s test proves an association of HPV 44 with diabetes because it only deals with discordant frequencies, and for this virus, the imbalance is large. This suggests that diabetes may be associated with a slightly increased likelihood of HPV 44 infection, but the effect was weak and likely influenced by the small sample size and low frequency of the virus.

## 4. Discussion

The results of the comparison between patients with and without type 2 diabetes (Table 2, Figure 1) demonstrate a higher frequency of viral infections in the diabetic group for all viruses analyzed, with statistically significant differences being found for EBV, HPV 42, and HPV 43. These results reflect differences between independent groups, but do not allow for a direct conclusion of an association at the individual level. Descriptive analysis of the distribution of diabetes status among virus-positive and virus-negative patients (Figure 3 and Figure 4) shows a similar trend, with a higher proportion of patients with diabetes being higher among the virus-positive subgroups. However, such distributions alone are not sufficient to assess an association, as they do not account for the structure of discordant categories.

Accordingly, McNemar’s test was applied, which allows for the analysis of the relationship between diabetes status and viral infection in the same patients (Table 3, Figure 2). After correction by the Holm–Bonferroni method, a statistically significant association with type 2 diabetes was found only for EBV and HPV 44, while the other viruses did not reach statistical significance. These associations remained statistically significant after Holm–Bonferroni correction for multiple comparisons.

Although HPV42 and HPV43 were more frequent in the diabetic group (Table 2), no statistically significant association between diabetes status and infection with these viruses was confirmed by the McNemar analysis after Holm–Bonferroni correction. This discrepancy reflects the fact that the χ^2^/Fisher tests and the McNemar test evaluate different aspects of the data: the former compare frequencies between independent groups, whereas the latter evaluates asymmetry between the ‘DM2 only’ and ‘V only’ categories within the same patient-level dataset. The lack of statistical significance in the McNemar analysis may additionally reflect the limited statistical power of the study and the increased risk of type II error associated with the small sample size.

A more detailed analysis of EBV and HPV 44 (Figure 3 and Figure 4) reveals a different degree of expression of the effect. In EBV, a clear distinction was observed between virus-positive and virus-negative patients in terms of diabetes status, whereas in HPV 44, the difference between subgroups was less pronounced, despite the presence of statistical significance.

The results are consistent with our previous study [5], in which a statistically significant co-infection between EBV and HPV 44 was found. This repeatability of statistically significant results in two independent analyses supports the presence of a specific pattern in which EBV and HPV 44 are characterized by both mutual association and a higher frequency in patients with type 2 diabetes. Given the small number of HPV44-positive cases in the present study (n = 5), these findings should be validated in larger cohorts.

Our study confirms and extends previous studies in this area. For example, Paradowski et al. [12] reported that patients with type 2 diabetes are associated with more advanced EBV-positive oropharyngeal squamous cell carcinoma (OPSCC) and significantly lower levels of anti-EBV antibodies. According to the authors, this fact may be associated with impaired antiviral immune control in patients with type 2 diabetes. In our study, the finding of a higher prevalence of EBV in patients with type 2 diabetes and advanced SCC of the external auricle is consistent with the idea that diabetes-related immune dysfunction facilitates viral persistence and oncogenesis. Hung et al. [16] demonstrated an association between prior HPV infection and risk of developing nasopharyngeal carcinoma (NPC), independent of diabetes, indicating an oncogenic role of HPV in head and neck cancer, although their study did not stratify study participants by diabetes status and did not include EBV infection. On the other hand, some authors [20,21,34] have linked T2DM to the risk and progression of head and neck cancer, identifying immune impairment and chronic inflammation as factors facilitating viral persistence, especially that of HPV, where DM2 has been identified as a prognostic factor [35]. Lo et al. [17] have highlighted the etiological role of HPV in keratinizing NPC type I (according to the WHO classification), distinct from the non-keratinizing subtypes associated with EBV, highlighting the heterogeneity of viral oncogenesis in head and neck cancer. Peng et al. [22] and Tseng et al. [23] have further documented diabetes as an independent risk factor for poorer prognosis and increased incidence of nasopharyngeal carcinoma and head and neck cancer. neck, which supports the clinical relevance of metabolic dysregulation in these malignancies. Cebioglu et al. [27] reviewed the molecular pathways linking diabetes to cancer susceptibility, including activation of viral oncogenes and oxidative stress, providing a conceptual framework for our findings.

The above facts could be provoked by the consequences associated with diabetes—metabolic changes, impaired glucose-insulin homeostasis and hyperglycemia, hormonal deregulation, insufficient detoxification with subsequent excessive production of reactive oxygen species (ROS), mitochondrial dysfunction with subsequent low energy production, insufficient repair capacity, and accumulation of damage to both chromosomal and mitochondrial DNA [27]. Also, hyperglycemia in diabetes causes dysfunction of the immune response, which fails to control the spread of invading pathogens in diabetic patients [27]. Dysfunction of the immune system in this case is associated with reduced production of IL-1beta, IL-2, IL-6, and IL-10. Interleukin-2 (IL-2) performs key functions during immune homeostasis through its effects on regulatory T (Treg) cells and the optimization of the fine-tuning of effector lymphocyte responses [36]. IL6 is important for the adaptive immune response as it induces antibody production and the development of effector T cells [37]. Reduced production of this cytokine leads to reduced production of INF-gamma and TNF-alpha by T cells [38]. Also, IL10 leads to down regulate T helper 1-type responses and suppression of the secretion and activation of IFN-γ [39]. All mechanisms of the immune system dysregulation listed so far lead to a disruption of the fine balance of the immune response, which makes diabetic patients more susceptible to infections [38].

The presence, proven by us, of viruses with oncogenic and/or oncomodulatory activity in patients with tumors of the external auricle on the one hand and type 2 diabetes on the other, complicates the clinical course of the diseases in these patients. The main viruses proven in our study are EBV and low-risk genotypes of HPV, which we believe are relevant to both diseases. EBV has co-evolved with our species for millions of years and is widely distributed in the human population as a lifelong and largely asymptomatic infection [40]. The main defense against EBV infection is T cell immunity, mainly CD8 and, to a lesser extent, CD4, and NK cells [40]. EBV, on the other hand, possesses various mechanisms for evading the immune response.

EBV evades the immune response by the use of three early lytic proteins, the synthesis of which could lead to immunodeficiency states. These proteins include BNLF2A, which binds to the TAP transporter, inhibiting the delivery of peptides to nascent HLA molecules [41,42,43], and BILF1, which binds to HLA I, disrupting the presentation of immunogenic complexes and enhancing lysosomal degradation of existing complexes from the surface [44,45]. BGLF5 reduces the expression of new HLA antigen as part of its general function to prevent the host immune response [46]. Proteins responsible for the latent state of EBV, such as EBNA and LMP1, stimulate cell proliferation and inhibit apoptosis, facilitating malignant transformation. Also, EBV microRNA (miR-BART2) reduces the expression of the NK-activating ligand MicB (Nachmani et al. 2009 [47]), and this is one way to evade the NK cell response [40]. Additionally, EBV, through the immediate early protein BZLF1, downregulates the expression of the class II transactivator CIITA [48]. The mechanisms for escaping the immune response in EBV also include the synthesis of virokines, which in turn further modulate the immune response.

There are several characteristic features in patients with type 2 diabetes. On the one hand, as described above, there is weakened cellular immunity. An additional factor is that, as a result of the clinical course of diabetes, the body reacts with chronic inflammation, which in turn leads to relatively slow tissue repair. The listed factors, acting together, could lead to reactivation of viruses, including EBV, a higher risk of tumor formation, and a more severe course. In our study, we assume that the EBV virus is rather a concomitant and oncomodulating factor, as diabetes, by weakening the immune system, rather affects the course and recovery from the tumor disease. From what has been described so far, we can assume and summarize: the disease, type 2 diabetes, weakens the immune system, which could lead to reactivation of latent infections, in our case, to reactivation of EBV. Reactivation of this virus may, in turn, modulate an oncogenic process, i.e., diabetes does not provoke tumor processes, but creates a favorable environment for their development. The combination of proven squamous cell carcinoma, type 2 diabetes, and the presence of EBV is more likely to have an impact on the prognosis of the disease and recovery from it.

In HPV, the situation is similar—immune response is mediated mainly by the two classes of T cells, CD4 and CD8, macrophages, and the cytokines IL-12, TNF-α, and IFN-γ [49].

Despite their designation as low-risk HPV 6, 11, 42, 44, and 70, they are found in invasive cervical cancer, anus [50], in the development of SCC [5,51], anal and perianal squamous cell carcinomas and high-grade intraepithelial lesions [52], oral squamous cell carcinoma and oral potentially malignant disorders [53]. This suggests an oncomodulatory effect [5] and/or mechanisms different from the classical carcinogenesis associated with high-risk HPV [53].

There are several mechanisms for escape from the immune response in HPV. The first mechanism is related to the characteristic replicative cycle of the virus. By infecting epithelial cells without achieving viremia, the inflammatory signal from the body is extremely weak. In fact, cell death, which can generate danger signals, is a prerequisite for inflammation. Thus, during most of the HPV infection cycle, there is little or no release of pro-inflammatory cytokines, which are important for the activation and migration of dendritic cells in the local environment, and the main signals necessary for immune responses in squamous epithelium are absent [50,54].

On the other hand, virus-specific proteins, mainly E6 and E7, inhibit interferon production and suppress signaling pathways involving Toll-like receptors and NF-kappa B. HPV infection leads to reduced production of MHC class 1 molecules and hence antigen presentation. This fact, in turn, makes the cells difficult to recognize by cytotoxic T lymphocytes. Another mechanism of escape from the immune response in these viruses is the suppression of apoptosis by degradation of p53, which is carried out by the E6 protein of the virus. Additionally, early HPV proteins E6 and E7 disrupt tumor suppressor pathways associated with Rb degradation and inactivation, leading to uncontrolled cell growth/replication [9,10]. Another mechanism is the chronicity of the infection by increasing the synthesis of a specific population of regulatory T cells.

We assume that in the case of proven low-risk HPV genotypes, which do not cause cancer by themselves, it is a “promoter” of carcinogenesis in diabetic patients. On the one hand, there are mechanisms for escape from the immune system by the virus; on the other hand, chronic inflammation, additional immune dysfunction, oxidative stress caused by glycosylated proteins, and difficult regeneration of the skin epithelium due to diabetes. In addition, we assume that the accumulation of ROS can lead to DNA damage and impaired reparative mechanisms. The combination of HPV and chronic inflammation due to diabetes can lead to increased cell proliferation and suppression of apoptosis. All of the above make the environment extremely favorable for virus persistence, the occurrence of lesions, and a worse prognosis of the tumor disease.

In connection with the above-mentioned disorders related to T cell function, due to the presence of diabetes, it is shown that EBV and HPV infections cannot be contained and controlled by the immune response and can develop to their full and ultimate extent, namely, to their oncogenic potential. The roles of EBV and HPV in head and neck oncogenesis are well established, with EBV being mainly implicated in T- and B-cell lymphoproliferative diseases and nasopharyngeal carcinoma [40,51,55], and HPV in oropharyngeal and other squamous cell carcinomas [52,56].

The identification in our study of a higher prevalence of EBV in patients with type 2 diabetes supports the hypothesis that diabetes-related immune impairment may reduce effective viral clearance or control, leading to persistent EBV infection and increased oncogenic potential [12], for the reasons described above. The observed association with HPV44, a low-risk HPV type, may reflect a broader susceptibility to HPV infections in patients with diabetes, although its oncogenic role, if any, is less clear compared with high-risk types. The lack of statistical significance for HPV42 and HPV43 after correction for multiple comparisons does not exclude the possibility of a real association, but may reflect the limited statistical power of the study and the increased risk of a type II error associated with the small sample size.

The biological interpretation of our results could be that diabetes mellitus creates a favorable environment for viral persistence and carcinogenesis through multiple interrelated mechanisms. Chronic hyperglycemia and insulin resistance induce systemic low-grade inflammation, characterized by increased proinflammatory cytokines such as interleukin-6 and tumor necrosis factor-alpha, which contribute to oxidative stress and genomic instability. These factors impair innate and adaptive immune responses, including neutrophil chemotaxis, macrophage antigen presentation, and T-cell activation, thereby reducing antiviral immunity critical for controlling latent infections such as EBV and HPV [12,19,20,21,22,23,27]. Hyperinsulinemia and increased insulin-like growth factor-1 (IGF-1) signaling further promote tumor proliferation, angiogenesis, and resistance to apoptosis through activation of the PI3K/Akt/mTOR pathway. These mechanisms, detailed in Wang et al. [20] and supported by Cebioglu et al. [27], provide a plausible pathophysiological basis for the observed higher viral load in DM2 patients and their poorer oncological prognosis, as reported by Paradowski et al. [12] and Peng et al. [22].

Everything stated so far shows that even low-risk HPV genotypes, such as 44, under these conditions—the presence of diabetes with its changes in immune reactivity, can have an impact on oncogenesis and contribute, together with other factors (UV, smoking, and alcohol), to the development of SCC in the area of the external ear.

The same applies to EBV, which is associated mainly with the development of B-cell malignancies, Burkitt Hodgkin T and NK cell lymphomas, post-transplant lymphoproliferative disease, epithelial tumors, and nasopharyngeal carcinoma [40,51], and in our case, due to the reasons stated above, it may exhibit oncomodulatory functions in the development of SCC in the area of the external ear.

Clinically, our findings highlight the importance of recognizing DM2 as a potential modifier of viral oncogenesis in head and neck tumors. The increased prevalence of EBV and some HPV types in diabetic patients may contribute to more aggressive tumor phenotypes and poorer prognosis, necessitating increased surveillance and tailored therapeutic approaches. Epidemiologically, the results are consistent with large cohort studies such as those conducted by Tseng et al. [23], which have documented an increased incidence of head and neck cancer in diabetic populations. Integrating metabolic control and antiviral strategies could potentially improve outcomes in this high-risk group. Furthermore, the significant association of HPV44 with DM2, despite its classification as low-risk, encourages further exploration of the spectrum of HPV genotypes associated with diabetic oncogenesis and the potential role of coinfection, as suggested by the observed patterns of coinfection with EBV.

### Limitations

The study has several limitations that affect the interpretation of the results. The relatively small sample size (n = 41) limits statistical power and generalizability. This limitation is particularly important for low-prevalence viruses such as HPV 44, for which only five positive cases were identified. In addition, small subgroup sizes and correction for multiple comparisons may have reduced the sensitivity to detect weaker associations and increased the risk of type II error. The sample size is small due to the relatively rare disease and the small population of the country. Our study is not intended to be a large-scale clinical trial. The cross-sectional design precludes causal inferences or conclusions regarding the temporal relationship between diabetes and viral infection. There was no adjustment for critical factors such as smoking and alcohol consumption, which are established risk factors for head and neck cancer [53,54,55,57,58,59], and the reason is that only one of the patients said that he smoked, but rarely, and none of them mentioned alcohol abuse. Thus, the indicated ones are not sufficient for statistical processing. No history has been taken of whether patients are taking metformin. Furthermore, the design with a single cohort from a single clinical center may reflect specific demographic or clinical characteristics that are not representative of broader populations. These limitations are consistent with those noted in comparative studies such as those conducted by Paradowski et al. [12] and Hung et al. [16], which also face challenges related to sample size, virus detection methods, and adjustment for predisposing factors.

## 5. Conclusions

In summary, the study provides new evidence for a significant association between type 2 diabetes mellitus and increased prevalence of EBV and some low-risk HPV genotypes in patients with head and neck tumors. The persistent associations between EBV and HPV44 after rigorous statistical adjustment underscore their potential biological and clinical significance in the oncogenic environment in the presence of type 2 diabetes. Our results are consistent with our previous study [5], which found a statistically significant co-infection between EBV and HPV 44. Low-risk HPV genotypes and EBV detected in a predominant percentage of samples from diabetic patients probably do not act as oncogenes, but as co-factors that, in the context of diabetes-induced immune dysregulation and chronic inflammation, promote tumor initiation and progression. The data suggest a possible synergistic model of action in patients with type 2 diabetes and the viruses under consideration, which, in this case, we assume act as co-factors in the development of squamous cell carcinoma of the auricle, probably through mechanisms involving viral persistence, chronic inflammation, and metabolism-induced oxidative stress. These findings contribute to the growing body of literature elucidating the interplay between metabolic disorders, viral infections, and cancer pathogenesis, highlighting the need for integrated clinical management strategies and further research to elucidate mechanistic pathways and therapeutic implications.

## Figures and Tables

**Table 1:** Association between type 2 diabetes and viral infection using McNemar analysis.

	Virus+	Virus−
DM2+	a	b
DM2−	c	d

+ means there is a virus or diabetes, − means there is no virus or diabetes. The frequencies in the cells in terms of the number of patients with the four combinations are indicated by lowercase Latin letters—a, b, c, and d.

**Table 2:** Prevalence of viral infections in patients with and without type 2 diabetes mellitus (DM2) and corresponding *p*-values from χ^2^ or Fisher’s exact test, depending on expected cell counts.

	DM2 (n = 27)		nDM2 (n = 14)		*p*-Value
	Number	%	Number	%	
HPV LR	25	92.60%	11	78.60%	0.317
HPV 6/11	22	81.50%	11	78.60%	1
HPV 42	21	77.80%	4	28.60%	0.006
HPV 43	16	59.30%	3	21.40%	0.048
HPV 44	4	14.80%	1	7.10%	0.645
HV	16	59.30%	2	14.30%	0.016
HSV-1	2	7.40%	1	7.10%	1
CMV	1	3.70%	0	0.00%	1
EBV	16	59.30%	1	7.10%	0.004

**Table 3:** Association between type 2 diabetes mellitus (DM2) and viral infections (HPV 6/11, 42, 43, 44, and EBV) based on McNemar’s test.

	HPV 6/11	HPV 42	HPV 43	HPV 44	EBV
DM2 and V	53.70%	51.20%	39.00%	9.80%	39.00%
DM2 only	12.20%	14.60%	26.80%	56.10%	26.80%
V only	26.80%	9.80%	7.30%	2.40%	2.40%
neither	7.30%	24.40%	26.80%	31.70%	31.70%
McNemar’s χ2	1.56	0.1	3.5	18.4	6.75
Adjusted *p* (Holm-Bonferroni)	0.423	0.752	0.184	<0.001	0.037

## Data Availability

The original contributions presented in this study are included in the article. Further inquiries can be directed to the corresponding author(s).

## Abbreviations

The following abbreviations are used in this manuscript:

BKPyV	BK polyomavirus
DM2	Diabetes Mellitus Type 2
DNA	Deoxyribonucleic acid
EBV	Epstein–Barr virus
HCMV	Human cytomegalovirus
HLA	Human Leukocyte Antigens
HSVs	Herpes simplex viruses
MHC	Major Histocompatibility Complex
ROS	Reactive Oxygen Species
SCC	Squamous cell carcinoma
OPSCC	oropharyngeal squamous cell carcinoma
NPC	nasopharyngeal carcinoma
HCMV	Human cytomegalovirus
